# The effects of regular dental scaling on the complications and mortality after stroke: a retrospective cohort study based on a real-world database

**DOI:** 10.1186/s12903-023-03178-6

**Published:** 2023-07-14

**Authors:** Li-Chin Sung, Chuen-Chau Chang, Chun-Chieh Yeh, Chia-Yen Lee, Chaur-Jong Hu, Yih-Giun Cherng, Ta-Liang Chen, Chien-Chang Liao

**Affiliations:** 1 Division of Cardiology, Department of Internal Medicine, School of Medicine, College of Medicine, Taipei Medical University, Taipei City, Taiwan; 2 Division of Cardiology, Department of Internal Medicine, Shuang Ho Hospital, Taipei Medical University, New Taipei City, Taiwan; 3 Taipei Heart Institute, Taipei Medical University, Taipei, Taiwan; 4 Department of Anesthesiology, School of Medicine, College of Medicine, Taipei Medical University, Taipei, Taiwan; 5 Anesthesiology and Health Policy Research Center, Taipei Medical University Hospital, Taipei, Taiwan; 6 Department of Anesthesiology, Taipei Medical University Hospital, Taipei, 11031 Taiwan; 7 Department of Surgery, China Medical University Hospital, Taichung, Taiwan; 8 Department of Surgery, University of Illinois, Chicago, United States of America; 9 Department of Dentistry, Taiwan Adventist Hospital, Taipei, Taiwan; 10 Department of Neurology, Shuang Ho Hospital, Taipei Medical University, New Taipei City, Taiwan; 11 Department of Anesthesiology, Shuang Ho Hospital, Taipei Medical University, New Taipei City, Taiwan; 12 Department of Anesthesiology, Wan Fang Hospital, Taipei Medical University, Taipei, Taiwan; 13 Research Center of Big Data and Meta-Analysis, Wan Fang Hospital, Taipei Medical University, Taipei, Taiwan; 14 School of Chinese Medicine, College of Chinese Medicine, China Medical University, Taichung, Taiwan

**Keywords:** Complications, Dental scaling, Mortality, Hospitalization, Stroke

## Abstract

**Background:**

Previous observational studies have shown that people with dental scaling (DS) had decreased risk of stroke. However, limited information is available on the association between DS and poststroke outcomes. The present study aimed to evaluate the effects of regular DS on the complications and mortality after stroke.

**Methods:**

We conducted a retrospective cohort study of 49,547 hospitalized stroke patients who received regular DS using 2010–2017 claims data of Taiwan’s National Health Insurance. Using a propensity-score matching procedure, we selected 49,547 women without DS for comparison. Multiple logistic regressions were used to calculate the odds ratios (ORs) and 95% confidence intervals (CIs) of poststroke complications and in-hospital mortality associated with regular DS.

**Results:**

Stroke patients with regular DS had significantly lower risks of poststroke pneumonia (OR 0.58, 95% CI 0.54–0.63), septicemia (OR 0.58, 95% CI 0.54–0.63), urinary tract infection (OR 0.68, 95% CI 0.66–0.71), intensive care (OR 0.81, 95% CI 0.78–0.84), and in-hospital mortality (OR 0.66, 95% CI 0.62–0.71) compared with non-DS stroke patients. Stroke patients with regular DS also had shorter hospital stays (p < 0.0001) and less medical expenditures (p < 0.0001) during stroke admission than the control group. Lower rates of poststroke adverse events in patients with regular DS were noted in both sexes, all age groups, and people with various types of stroke.

**Conclusion:**

Stroke patients with regular DS showed fewer complications and lower mortality compared with patients had no DS. These findings suggest the urgent need to promote regular DS for this susceptible population of stroke patients.

**Supplementary Information:**

The online version contains supplementary material available at 10.1186/s12903-023-03178-6.

## Background

Stroke was considered one of the leading causes of death worldwide, and the number of stroke deaths increased 41.4% between 1990 and 2017 with approximately 5.5 million stroke deaths per year [1, 2]. The high lifetime prevalence and economic burden to society implicated the importance of stroke prevention and poststroke care [3]. Stroke in many patients is caused by atherosclerosis, which is a sustained, dynamic disease hallmarked by chronic inflammation in the vasculature. Previous studies have demonstrated that microbial infections accelerate vascular inflammation, suggesting that chronic infection also plays an important role in the development of atherosclerosis [4, 5]. Although the epidemiology, pathogenesis, prevention, and treatment of stroke have been well established [6–8], the investigation of protective factors remains an area of research interest.

Oral hygiene is crucial for general health, with periodontitis being one of the most prevalent chronic inflammatory diseases worldwide [9, 10]. Several pro-inflammatory cytokines, such as interleukin (IL)-1, IL-6, C-reactive protein, fibrinogen and serum amyloid A, have been shown to be elevated in patients with periodontitis [11, 12]. Additionally, major disease-causing bacterial strains of periodontitis are also present in atherosclerotic plaques [13]. Many observational studies and meta-analysis have reported that patients with periodontitis have increased risk for stroke [9, 11, 14, 15]. In contrast, oral hygiene care such as tooth brushing at least twice a day and regular professional dental cleaning (scaling and root planing) reduced the risk of future Meniere’s disease, rheumatoid arthritis, stroke, and cardiovascular events in general population [16–19].

Dental scaling (DS), meaning deep tooth cleaning with removal of dental plaque and calculus, was associated with a decreased risk for stroke, irrespective of the presence of periodontitis or not [9–11]. Patients with periodontitis are more at risk for pyogenic liver abscess than controls, particularly when they have no DS [20]. DS was significantly associated with lower risks of progression and mortality to end-stage renal disease in patients with chronic kidney disease [21]. Both dental scaling and intensive treatment for periodontal disease are associated with a lower risk of further ischaemic stroke events [9]. Regular DS once a year or more was shown to reduce cardiovascular risk by 14% in one population-based cohort study [19]. However, limited evidence is available on the association between regular DS and poststroke outcomes. Thus, we conducted a nationwide matched study using research data from Taiwan’s National Health Insurance to investigate the effects of regular DS on the outcomes after stroke.

## Methods

### Source of data

We mined the research database of Taiwan’s National Health Insurance to conduct this study. The National Health Insurance program has been implemented in Taiwan since March 1995. The research database of the National Health Insurance collected all medical reimbursement claims since 1996 and is available to researchers with beneficiaries’ identification scrambled to protect privacy. The available information of this database includes sex, birth date, diagnoses, examinations, prescriptions, treatments, and medical expenditures related to outpatient visits, emergency care, and hospitalization. The reliability of this database has been well accepted by important scientific journals worldwide [22, 23]. This study was approved by the institutional review board of Taipei Medical University (TMU-JIRB-202203134; TMU-JIRB-202006057).

### Study design

We examined medical claims and identified 378,006 first-ever stroke patients aged ≥ 20 years who underwent inpatient care by a neurologist (defined as patients with a physician’s primary diagnosis of stroke as well as hospitalization for > 1 day) in 2010–2017. After excluding ineligible study subjects (those that received irregular DS), we identified 62,174 stroke patients who had received regular DS (defined as receiving four DS within the previous 24 months before stroke admission) and 178,603 stroke patients who had no DS (defined as receiving no DS within the previous 24 months before stroke admission). Each patient who underwent inpatient care for stroke and received DS was randomly matched to a patient without DS who underwent inpatient care for stroke using a propensity score matched-pair procedure to adjust for sociodemographics, characteristics of stroke hospitalization, and coexisting medical conditions. After propensity score matching (case-control ratio, 1:1), 49,547 patients with and 49,547 without prestroke regular DS were included.

### Measures and definitions

According to the recommendation of Taiwan’s Ministry of Health and Welfare, people are encouraged to receive DS every six months, and the National Health Insurance Administration also provides corresponding medical payment for DS. Therefore, the people in Taiwan receive free DS every six months. In this study, we defined DS as people who received 4 times of DS services within 24 months before stroke admission. Irregular DS was defined as people who had 1, 2, or 3 visits of DS within 24 months before stroke admission. Low-income status is defined as having a low income within 2 years before the first-ever stroke admission. According to the regulations from the Agency of National Health Insurance in Taiwan, people with a low-income status were qualified to have the registration fee and medical copayment waived when receiving outpatient, emergency, and inpatient medical care.

The International Classification of Diseases, Ninth Revision, Clinical Modification (ICD-9-CM) was used to define the physicians’ diagnoses. Based on our previous studies [22, 23], preoperative medical conditions determined from the medical claims for the 24-month preoperative period included hypertension (ICD-9-CM 401–405), diabetes (ICD-9-CM 250), mental disorders (ICD-9-CM 290–319), ischemic heart disease (ICD-9-CM 410–414), chronic obstructive pulmonary disease (ICD-9- CM 491,492, 496), hyperlipidemia (ICD-9-CM 272.0, 272.1, 272.2, 272.4), heart failure (ICD-9-CM 428), Parkinson’s disease (ICD-9-CM 332), and liver cirrhosis (ICD-9-CM 571.2, 571.5,571.6). Renal dialysis was defined by administration codes (D8 and D9). Postoperative pneumonia (ICD-9-CM 480–486), septicemia (ICD-9-CM 038 and 998.5), urinary tract infection (ICD-9-CM 599.0), and in-hospital mortality during the index stroke admission were considered the study’s primary outcomes. Admission to the intensive care unit, hospital stay length, and medical expenditures during the index stroke admission were also compared between patients with and without prestroke regular DS.

### Statistical analysis

We used propensity score-matched pair analysis to determine the associations between regular DS and poststroke outcomes. A nonparsimonious multivariable logistic regression model was used to estimate a propensity score for stroke patients who did or did not receive regular DS. Clinical significance guided the initial choice of covariates in this model and included age, sex, low income, types of stroke, hypertension, diabetes, mental disorders, ischemic heart disease, chronic obstructive pulmonary disease, hyperlipidemia, heart failure, Parkinson’s disease, liver cirrhosis, and renal dialysis. We matched stroke patients with regular DS to non-DS patients using a greedy matching algorithm (without replacement) with a caliper width of 0.2 SD of the log odds of the estimated propensity score. Categorical variables were summarized using frequency (percentage) and were compared between DS and non-DS stroke patients using chi-square tests. Continuous variables were summarized using the mean ± standard deviation and were compared using t tests. Logistic regressions were used to calculate the odds ratios (ORs) and 95% confidence intervals (CIs) of poststroke outcomes associated with regular DS. Additional sensitivity analyses stratified by age, sex, number of medical conditions and types of stroke were also performed to examine poststroke adverse events in patients with and without DS within these strata.

## Results

The baseline characteristics of 62,174 stroke patients with regular DS and 178,603 stroke patients without DS are shown in Table 1. After matching by propensity-score, no significant differences in age, sex, low-income status, types of stroke, hypertension, diabetes, mental disorders, ischemic heart disease, chronic obstructive pulmonary disease, hyperlipidemia, heart failure, Parkinson’s disease, liver cirrhosis, and renal dialysis were identified between stroke patients with and without regular DS (Table 2).

**Table 1:** Characteristics of hospitalized stroke patients with and without dental scaling (before matching)

	No DS (N = 178,603)		DS (N = 62,174)		p-value
Sex	n	(%)	n	(%)	< 0.0001
Female	71,597	(40.1)	26,110	(42.0)	
Male	107,006	(59.9)	36,064	(58.0)	
Age, years					< 0.0001
20–29	413	(0.2)	718	(1.2)	
30–39	2913	(1.6)	2111	(3.4)	
40–49	11,012	(6.2)	5337	(8.6)	
50–59	27,145	(15.2)	12,803	(20.6)	
60–69	36,658	(20.5)	16,759	(27.0)	
70–79	47,608	(26.7)	15,458	(24.9)	
≥ 80	52,854	(29.6)	8988	(14.5)	
Low income					< 0.0001
No	168,344	(94.3)	60,352	(97.1)	
Yes	10,259	(5.7)	1822	(2.9)	
Number of hospitalizations					< 0.0001
0	120,193	(67.3)	44,405	(71.4)	
1	33,996	(19.0)	11,253	(18.1)	
2	12,696	(7.1)	3595	(5.8)	
≥ 3	11,718	(6.6)	2921	(4.7)	
Number of emergency visits					< 0.0001
0	86,872	(48.6)	30,162	(48.5)	
1	47,273	(26.5)	17,225	(27.7)	
2	21,822	(12.2)	7564	(12.2)	
≥ 3	22,636	(12.7)	7223	(11.6)	
Medical conditions					
Hypertension	72,393	(40.5)	25,898	(41.7)	< 0.0001
Diabetes	43,409	(24.3)	14,536	(23.4)	< 0.0001
Mental disorders	33,373	(18.7)	13,692	(22.0)	< 0.0001
Ischemic heart disease	20,083	(11.2)	8756	(14.1)	< 0.0001
COPD	19,587	(11.0)	7239	(11.6)	< 0.0001
Hyperlipidemia	8165	(4.6)	5188	(8.3)	< 0.0001
Heart failure	10,035	(5.6)	2158	(3.5)	< 0.0001
Parkinson’s disease	6231	(3.5)	1923	(3.1)	< 0.0001
Liver cirrhosis	4401	(2.5)	1777	(2.9)	< 0.0001
Renal dialysis	5193	(2.9)	1263	(2.0)	< 0.0001
Types of stroke					< 0.0001
Subarachnoid hemorrhage	4838	(2.7)	2426	(3.9)	
ICH	32,655	(18.3)	8260	(13.3)	
Other and unspecified ICH	5373	(3.0)	1867	(3.0)	
Occlusion of precerebral arteries	5165	(2.9)	2339	(3.8)	
Occlusion of cerebral arteries	107,002	(59.9)	32,314	(52.0)	
Transient cerebral ischemia	18,459	(10.3)	10,855	(17.5)	
Acute, but ill-defined CVD	2127	(1.2)	1461	(2.4)	
Other and ill-defined CVD	2984	(1.7)	2652	(4.3)	

CVD, cerebrovascular disease; COPD, chronic obstructive pulmonary disease; DS, dental scaling; ICH, intracerebral hemorrhage.

**Table 2:** Characteristics of hospitalized stroke patients with and without dental scaling (after matching)

	No DS (N = 49,547)		DS (N = 49,547)		p-value
Sex	n	(%)	n	(%)	1.0000
Female	19,542	(39.4)	19,542	(39.4)	
Male	30,005	(60.6)	30,005	(60.6)	
Age, years					1.0000
20–29	260	(0.5)	260	(0.5)	
30–39	1327	(2.7)	1327	(2.7)	
40–49	3977	(8.0)	3977	(8.0)	
50–59	9946	(20.1)	9946	(20.1)	
60–69	13,482	(27.2)	13,482	(27.2)	
70–79	12,936	(26.1)	12,936	(26.1)	
≥ 80	7619	(15.4)	7619	(15.4)	
Low income					1.0000
No	48,670	(98.2)	48,670	(98.2)	
Yes	877	(1.8)	877	(1.8)	
Number of hospitalizations					1.0000
0	37,908	(76.5)	37,908	(76.5)	
1	8116	(16.4)	8116	(16.4)	
2	2010	(4.1)	2010	(4.1)	
≥ 3	1513	(3.1)	1513	(3.1)	
Number of emergency visits					1.0000
0	26,136	(52.8)	26,136	(52.8)	
1	13,858	(28.0)	13,858	(28.0)	
2	5212	(10.5)	5212	(10.5)	
≥ 3	4341	(8.8)	4341	(8.8)	
Medical conditions					
Hypertension	20,546	(41.5)	20,546	(41.5)	1.0000
Diabetes	10,907	(22.0)	10,907	(22.0)	1.0000
Mental disorders	8569	(17.3)	8569	(17.3)	1.0000
Ischemic heart disease	5024	(10.1)	5024	(10.1)	1.0000
COPD	4262	(8.6)	4262	(8.6)	1.0000
Hyperlipidemia	2511	(5.1)	2511	(5.1)	1.0000
Heart failure	1048	(2.1)	1048	(2.1)	1.0000
Parkinson’s disease	862	(1.7)	862	(1.7)	1.0000
Liver cirrhosis	649	(1.3)	649	(1.3)	1.0000
Renal dialysis	459	(0.9)	459	(0.9)	1.0000
Types of stroke					1.0000
Subarachnoid hemorrhage	1693	(3.4)	1693	(3.4)	
ICH	6919	(14.0)	6919	(14.0)	
Other and unspecified ICH	1170	(2.4)	1170	(2.4)	
Occlusion of precerebral arteries	1422	(2.9)	1422	(2.9)	
Occlusion of cerebral arteries	29,146	(58.8)	29,146	(58.8)	
Transient cerebral ischemia	7472	(15.1)	7472	(15.1)	
Acute, but ill-defined CVD	608	(1.2)	608	(1.2)	
Other and ill-defined CVD	1117	(2.3)	1117	(2.3)	

CVD, cerebrovascular disease; COPD, chronic obstructive pulmonary disease; DS, dental scaling; ICH, intracerebral hemorrhage.

Compared with non-DS controls (Table 3), stroke patients who received regular DS previously had lower risks of poststroke pneumonia (OR 0.58, 95% CI 0.54–0.63), septicemia (OR 0.58, 95% CI 0.54–0.63), urinary tract infection (OR 0.68, 95% CI 0.66–0.71), intensive care (OR 0.81, 95% CI 0.78–0.84), and in-hospital mortality (OR 0.66, 95% CI 0.62–0.71). Hospital stay length (9.3 ± 8.8 vs. 10.2 ± 9.3 days, P < 0.0001) and medical expenditure (2113 ± 3209 vs. 2233 ± 3198 US dollars, P < 0.0001) during the index stroke admission were also lower in patients who received regular DS compared to non-DS controls. The adverse outcome in stroke patients with and without dental scaling (before matching) was showed in Table S1. In Table 4, the stratification analysis showed that receiving regular DS was associated with reduced poststroke adverse events (including pneumonia, septicemia, urinary tract infection, and mortality) among men (OR 0.57, 95% CI 0.55–0.60), women (OR 0.65, 95% CI 0.62–0.68), patients aged 20–49 years (OR 0.49, 95% CI 0.44–0.55), patients aged 50–59 years (OR 0.56, 95% CI 0.52–0.61), patients aged 60–69 years (OR 0.61, 95% CI 0.57–0.65), patients aged 70–79 years (OR 0.62, 95% CI 0.59–0.66), and patients aged ≥ 80 years (OR 0.68, 95% CI 0.63–0.73). Subgroup analysis showed that regular DS lowered the risk of poststroke adverse events in patients with subarachnoid hemorrhage (OR 0.71, 95% CI 0.61–0.81), intracerebral hemorrhage (OR 0.65, 95% CI 0.61–0.70), other and unspecified intracerebral hemorrhage (OR 0.56, 95% CI 0.46–0.68), occlusion of precerebral arteries (OR 0.49, 95% CI 0.40–0.59), occlusion of cerebral arteries (OR 0.58, 95% CI 0.56–0.61), transient cerebral ischemia (OR 0.69, 95% CI 0.62–0.78), acute but ill-defined cerebrovascular disease (OR 0.33, 95% CI 0.22–0.49), and other ill-defined cerebrovascular disease (OR 0.56, 95% CI 0.42–0.75). Table S2 showed the stratified analysis for stroke patients with dental scaling associated with adverse events by medical conditions.

**Table 3:** Adverse outcomes of stroke patients with and without dental scaling

	No DS (N = 49,547)		DS (N = 49,547)		Risk of outcomes	
Post-stroke outcomes	Events	%	Events	%	OR	(95% CI)^a^
30-day in-hospital mortality	2254	4.6	1556	3.1	0.66	(0.62–0.71)
Pneumonia	5402	10.9	3343	6.8	0.58	(0.55–0.60)
Septicemia	1788	3.6	1060	2.1	0.58	(0.54–0.63)
Urinary tract infection	5893	11.9	4266	8.6	0.68	(0.66–0.71)
Stay in intensive care unit	13,861	28.0	12,464	25.2	0.81	(0.78–0.84)
Medical expenditure, USD^b^	2233 ± 3198		2113 ± 3209		P < 0.0001	
Length of hospital stay, days^b^	10.2 ± 9.3		9.3 ± 8.8		P < 0.0001	

CI, confidence interval; DS, dental scaling; OR, odds ratio.

*Adjusted for all covariates listed in Table 1.

†Mean ± SD; regular dental scaling was associated with length of hospital stays (beta = -0.84, P < 0.0001) and medical expenditure (beta = -119.8, P < 0.0001) after adjusted all covariates listed in Table 2 in the multiple linear regressions.

**Table 4:** The stratified analysis of dental scaling and post-stroke adverse events by age, sex, and types of stroke (N = 99,094)

			Adverse events*			
		n	Events	Rate, %	OR	(95% CI)†
Female	No DS	19,542	5521	28.3	1.00	(reference)
	DS	19,542	4089	20.9	0.65	(0.62–0.68)
Male	No DS	30,005	6564	21.9	1.00	(reference)
	DS	30,005	4345	14.5	0.57	(0.55–0.60)
Age 20–49 years	No DS	5564	1136	20.4	1.00	(reference)
	DS	5564	664	11.9	0.49	(0.44–0.55)
Age 50–59 years	No DS	9946	1894	19.0	1.00	(reference)
	DS	9946	1220	12.3	0.56	(0.52–0.61)
Age 60–69 years	No DS	13,482	2681	19.9	1.00	(reference)
	DS	13,482	1823	13.5	0.61	(0.57–0.65)
Age 70–79 years	No DS	12,936	3512	27.2	1.00	(reference)
	DS	12,936	2488	19.2	0.62	(0.59–0.66)
Age ≥ 80 years	No DS	7619	2862	37.6	1.00	(reference)
	DS	7619	2239	29.4	0.68	(0.63–0.73)
Subarachnoid hemorrhage	No DS	1693	731	43.2	1.00	(reference)
	DS	1693	595	35.1	0.71	(0.61–0.81)
ICH	No DS	6919	2907	42.0	1.00	(reference)
	DS	6919	2235	32.3	0.65	(0.61–0.70)
Other and unspecified ICH	No DS	1170	347	29.7	1.00	(reference)
	DS	1170	227	19.4	0.56	(0.46–0.68)
Occlusion of precerebral arteries	No DS	1422	350	24.6	1.00	(reference)
	DS	1422	199	14.0	0.49	(0.40–0.59)
Occlusion of cerebral arteries	No DS	29,146	6664	22.9	1.00	(reference)
	DS	29,146	4437	15.2	0.58	(0.56–0.61)
Transient cerebral ischemia	No DS	7472	836	11.2	1.00	(reference)
	DS	7472	610	8.2	0.69	(0.62–0.78)
Acute, but ill-defined CVD	No DS	608	107	17.6	1.00	(reference)
	DS	608	43	7.1	0.33	(0.22–0.49)
Other and ill-defined CVD	No DS	1117	143	12.8	1.00	(reference)
	DS	1117	88	7.9	0.56	(0.42–0.75)

CI, confidence interval; COPD, chronic obstructive pulmonary disease; CVD, cerebrovascular disease; DS, dental scaling; ICH, intracerebral hemorrhage; OR, odds ratio.

*Adverse events included with 30-day in-hospital mortality, pneumonia, septicemia, UTI.

†Adjusted for all covariates listed in Table 2.

Table S3 showed the adverse outcomes after stroke in patients with and without irregular DS (having 1, 2, or 3 visits of DS). Irregular DS was associated with reduced risks of post-stroke pneumonia (OR 0.75, 95% CI 0.71–0.78), septicemia (OR 0.71, 95% CI 0.66–0.77), urinary tract infection (OR 0.85, 95% CI 0.81–0.89), and 30-day in-hospital mortality (OR 0.88, 95% CI 0.84–0.93).

## Discussion

In this retrospective cohort study matched by propensity score, we found that stroke patients with regular DS had lower risks of poststroke pneumonia, septicemia, urinary tract infection, intensive care, and in-hospital mortality compared with those that did not receive DS. The medical expenditure and length of hospital stay were also lower in the stroke patients with DS compared to the control group. The association between DS and reduced poststroke adverse events was significant in both genders, every age group, and all stroke subtypes.

Factors associated with stroke and poststroke outcomes that were identified in previous studies include sex, age, hypertension, diabetes, mental disorders, ischemic heart disease, chronic obstructive pulmonary disease, hyperlipidemia, atrial fibrillation, heart failure, liver cirrhosis, dialysis, and Parkinson’s disease [6–8]. To avoid bias when investigating the relationships between DS and poststroke outcomes, we used propensity-score matching to adjust these potential confounding factors. We also considered socioeconomic status, number of hospitalizations and emergency visits as potential confounding factors in the multivariable regression models.

One case-control study reported that periodontal inflammation was associated with poor functional prognosis in patients diagnosed with minor stroke [24]. In another cohort study, systematic oral hygiene care was associated with decreased odds of hospital-acquired pneumonia in patients with acute-subacute stroke [25]; however, these studies had a relatively small sample size, limited types of stroke (ischemic or hemorrhagic) and lacked important prognostic implications (e.g., mortality). Our study correlated with the aforementioned reports in that maintenance of oral hygiene by DS is beneficial in post-stroke patients. Furthermore, our results demonstrated that DS has more beneficial effects on health outcomes in patients with stroke, which is rarely present in the literature.

Poor dentition increases the risk of bacteremia and the levels of serum IL-1 and IL-6 [11, 12, 26, 27]. Prior evidence has shown that oral bacterial pathogens may be identified in atherothrombotic tissues [13, 27]. Poor oral hygiene is also associated with a continuous infection and systemic inflammation, which could represent the underlying mechanism that links oral health and stroke. Routine dental examinations help patients identify dental problems and resolve them early. The Taiwan’s National Health Insurance program enrolled nearly all residents and provided dental checkups and scaling to all beneficiaries once per six months [28]. Previous studies found that these inflammatory biomarkers significantly decreased after the improvement of oral hygiene [11, 29]. Infectious complications commonly occurred after stroke and affect approximately 15-30% of stroke patients [30]. Poststroke infections directly contribute to high mortality in stroke patients [31–34]. We suggest that regular DS is beneficial in reducing inflammation, infectious complications and subsequent mortality after stroke [9, 13, 24, 25].

The beneficial effects of DS on poststroke outcomes could be partly explained by the following reasons. Firstly, the brain central nervous system and the immune system are closely linked via the hypothalamic-pituitary-adrenal axis and the sympathetic nervous system [30]. The mechanism of poststroke immunosuppression and immune exhaustion may increase the risk of infection [30–33, 35, 36]. The oral cavity serves as an endogenous reservoir for gut microbial strains [37], which intensifies the complex pathways to poststroke infection [32]. Receiving DS is helpful in reducing systemic inflammation [9, 13], gut dysbiosis, dissemination and subsequent infections in an immunosuppressive state [25, 38]. Secondly, people with tooth loss have increased risk of stroke mortality [39]. Tooth loss is also associated with pneumonia mortality [40]. Because regular DS is effective in maintaining functional oral intake and preventing tooth loss [28], we hypothesize that regular DS is beneficial in poststroke infections and mortality. Thirdly, some evidence indicates that poor oral hygiene may increase the risk of poststroke pneumonia [41]. In addition, patients who received regular DS are more likely to have better health awareness, socioeconomic status, family support and ambulatory independence [42–45], which are considered potential determinants of poststroke outcomes [46].

Billions of bacteria live inside our mouths at any given time, and many of these bacteria build up as plaque that causes tooth decay (cavities) and gingivitis, which can lead to periodontal disease. For a healthy oral cavity, the prevention of stroke, and improvement of poststroke outcomes, we recommend that people practice good oral hygiene. Teeth brushing, using antimicrobial mouthwash, and dental flossing every day, in addition to receiving dental scaling at least once per six months helps to reduce inflammation, atherosclerosis, stroke, and poststroke complications and mortality.

Our study has some limitations. Firstly, information regarding the participants’ detailed socioeconomics, lifestyle (such as smoking, alcohol consumption, physical activity), and biochemical laboratory measures were unavailable in the research data of Taiwan’s National Health Insurance. Future work can aim to include information on smoking habits, which affects both periodontal health and pulmonary conditions (pneumonia, etc.). Secondly, we could not consider the severity of stroke in this study because clinical data of brain infarct size, location, and the risk scores (such as National Institutes of Health Stroke Scale, modified Rankin scale or Barthel index) were not available in the current insurance database. Thirdly, we could not evaluate the protective effects of DS against specific oral pathogens because we had no information regarding the detailed dental data. Fourthly, we are unable to determine how well patients were able to maintain good daily oral hygiene habits, such as brushing twice a day with fluoridated toothpaste for 2 min, using floss, interproximal brushes, etc. In addition, no information was available regarding the self-paid (insurance-uncovered) dental medical services. Finally, residual confounding bias may exist in our study, although we attempted to control for potential confounders to the best of our ability.

In conclusion, the current study demonstrated that DS is associated with a reduced risk of poststroke complications and mortality. Our study suggests that the maintenance of oral hygiene by receiving regular DS (at least two times per year) may be an efficient way to manage cerebrovascular health. Further prospective studies are encouraged to confirm a causal relationship and potential biological mechanisms underlying the protective effects of DS for stroke patients.

## Electronic supplementary material

Below is the link to the electronic supplementary material.

Supplementary Material 1

## Data Availability

The datasets used and/or analyzed during the current study available from the Ministry of Health and Welfare. Interested researchers can obtain the data through formal application to the Health and Welfare Data Science Center, Department of Statistics, Ministry of Health and Welfare, Taiwan (http://dep.mohw.gov.tw/DOS/np-2497-113.html) and contact the agency with email (stpeicih@mohw.gov.tw).

## Abbreviations

CI: Confidence interval
ICD-9-CM: International Classification of Diseases, 9th Revision, Clinical Modification
OR: Odds ratio
