# Supplementary material for: The effects of regular dental scaling on the complications and mortality after stroke: a retrospective cohort study based on a real-world database
Source: BMC Oral Health. 2023 Jul 14;23:487. doi: 10.1186/s12903-023-03178-6 (PMC10349500; doi:10.1186/s12903-023-03178-6)
Supplement: Supplementary file 1 — Supplementary Material 1 [file 12903_2023_3178_MOESM1_ESM.doc]

| **Table S1. Adverse outcomes of stroke patients with and without dental scaling (before matching)** | | | | | | |
| --- | --- | --- | --- | --- | --- | --- |
|  | No DS (N=178603) | | DS (N=62174) | | Risk of outcomes | |
| Post-Stroke outcomes | Events | % | Event | % | OR | (95% CI)^a^ |
| 30-day in-hospital mortality | 10040 | 5.6 | 1984 | 3.2 | 0.61 | (0.64-0.71) |
| Pneumonia | 24597 | 13.8 | 4173 | 6.7 | 0.59 | (0.57-0.61) |
| Septicemia | 8131 | 4.6 | 1356 | 2.2 | 0.60 | (0.57-0.64) |
| Urinary tract infection | 26727 | 15.0 | 5284 | 8.5 | 0.66 | (0.64-0.68) |
| Stay in intensive care unit | 57271 | 32.1 | 15843 | 25.5 | 0.80 | (0.78-0.82) |
| Medical expenditure, USD^b^ | 2381±3237 | | 2179±3313 | | p<0.0001 | |
| Length of hospital stay, days^b^ | 11.1±9.8 | | 9.1±8.7 | | p<0.0001 | |
| CI = confidence interval; DS = dental scaling; OR = odds ratio.  ^a^ Adjusted for all covariates listed in Table 1.  ^b^Mean±SD; regular dental scaling was associated with length of hospital stays (beta = -1.11, P<0.0001) and medical expenditure (beta = -37.1 P<0.0001) after adjusted all covariates listed in Table 1 in the multiple linear regressions. | | | | | | |

| **Table S2. The stratified analysis for stroke patients with dental scaling associated with adverse events by medical conditions (N=99,094)** | | | | | | |
| --- | --- | --- | --- | --- | --- | --- |
|  | |  | Adverse events^a^ | | | |
|  |  | n | Events | Rate, % | OR | (95% CI)^b^ |
| 0 hospitalization | No DS | 37908 | 8505 | 22.4 | 1.00 | (reference) |
|  | DS | 37908 | 5774 | 15.2 | 0.59 | (0.57-0.62) |
| 1 hospitalization | No DS | 8116 | 2306 | 28.4 | 1.00 | (reference) |
|  | DS | 8116 | 1662 | 20.5 | 0.62 | (0.58-0.67) |
| 2 hospitalization | No DS | 2010 | 656 | 32.6 | 1.00 | (reference) |
|  | DS | 2010 | 532 | 26.5 | 0.73 | (0.63-0.84) |
| ≥3 hospitalizations | No DS | 1513 | 618 | 40.9 | 1.00 | (reference) |
|  | DS | 1513 | 466 | 30.8 | 0.62 | (0.53-0.73) |
| 0 emergency visit | No DS | 26136 | 5970 | 22.8 | 1.00 | (reference) |
|  | DS | 26136 | 4089 | 15.7 | 0.60 | (0.57-0.63) |
| 1 emergency visit | No DS | 13858 | 3328 | 24.0 | 1.00 | (reference) |
|  | DS | 13858 | 2243 | 16.2 | 0.58 | (0.55-0.62) |
| 2 emergency visit | No DS | 5212 | 1361 | 26.1 | 1.00 | (reference) |
|  | DS | 5212 | 1046 | 20.1 | 0.69 | (0.62-0.75) |
| ≥3 emergency visits | No DS | 4341 | 1426 | 32.9 | 1.00 | (reference) |
|  | DS | 4341 | 1056 | 24.3 | 0.63 | (0.57-0.69) |
| No hypertension | No DS | 29001 | 7116 | 24.5 | 1.00 | (reference) |
|  | DS | 29001 | 4815 | 16.6 | 0.58 | (0.56-0.61) |
| Hypertension | No DS | 20546 | 4969 | 24.2 | 1.00 | (reference) |
|  | DS | 20546 | 3619 | 17.6 | 0.64 | (0.61-0.68) |
| No diabetes | No DS | 38640 | 9299 | 24.1 | 1.00 | (reference) |
|  | DS | 38640 | 6502 | 16.8 | 0.61 | (0.59-0.63) |
| Diabetes | No DS | 10907 | 2786 | 25.5 | 1.00 | (reference) |
|  | DS | 10907 | 1932 | 17.7 | 0.60 | (0.56-0.64) |
| No hyperlipidemia | No DS | 47036 | 11614 | 24.7 | 1.00 | (reference) |
|  | DS | 47036 | 8102 | 17.2 | 0.61 | (0.59-0.63) |
| Hyperlipidemia | No DS | 2511 | 471 | 18.8 | 1.00 | (reference) |
|  | DS | 2511 | 332 | 13.2 | 0.64 | (0.54-0.75) |
| No mental disorders | No DS | 40978 | 9668 | 23.6 | 1.00 | (reference) |
|  | DS | 40978 | 6708 | 16.4 | 0.61 | (0.58-0.63) |
| Mental disorders | No DS | 8569 | 2417 | 28.2 | 1.00 | (reference) |
|  | DS | 8569 | 1726 | 20.1 | 0.61 | (0.57-0.66) |
| No ischemic heart disease | No DS | 44523 | 10775 | 24.2 | 1.00 | (reference) |
|  | DS | 44523 | 7471 | 16.8 | 0.60 | (0.58-0.62) |
| Ischemic heart disease | No DS | 5024 | 1310 | 26.1 | 1.00 | (reference) |
|  | DS | 5024 | 963 | 19.2 | 0.65 | (0.59-0.72) |
| No heart failure | No DS | 48499 | 11671 | 24.1 | 1.00 | (reference) |
|  | DS | 48499 | 8120 | 16.7 | 0.61 | (0.59-0.63) |
| Heart failure | No DS | 1048 | 414 | 39.5 | 1.00 | (reference) |
|  | DS | 1048 | 314 | 30.0 | 0.64 | (0.53-0.77) |
| No liver cirrhosis | No DS | 48898 | 11928 | 24.4 | 1.00 | (reference) |
|  | DS | 48898 | 8322 | 17.0 | 0.61 | (0.59-0.63) |
| Liver cirrhosis | No DS | 649 | 157 | 24.2 | 1.00 | (reference) |
|  | DS | 649 | 112 | 17.3 | 0.62 | (0.46-0.82) |
| No COPD | No DS | 45285 | 10880 | 24.0 | 1.00 | (reference) |
|  | DS | 45285 | 7499 | 16.6 | 0.60 | (0.58-0.62) |
| COPD | No DS | 4262 | 1205 | 28.3 | 1.00 | (reference) |
|  | DS | 4262 | 935 | 21.9 | 0.69 | (0.62-0.77) |
| No renal dialysis | No DS | 49088 | 11929 | 24.3 | 1.00 | (reference) |
|  | DS | 49088 | 8306 | 16.9 | 0.61 | (0.59-0.59) |
| Renal dialysis | No DS | 459 | 156 | 34.0 | 1.00 | (reference) |
|  | DS | 459 | 128 | 27.9 | 0.73 | (0.55-0.98) |
| No Parkinson’s disease | No DS | 48685 | 11794 | 24.2 | 1.00 | (reference) |
|  | DS | 48685 | 8198 | 16.8 | 0.61 | (0.59-0.63) |
| Parkinson’s disease | No DS | 862 | 291 | 33.8 | 1.00 | (reference) |
|  | DS | 862 | 236 | 27.4 | 0.73 | (0.59-0.90) |
| CI = confidence interval; COPD = chronic obstructive pulmonary disease; DS = dental scaling; ICH, intracerebral hemorrhage; OR = odds ratio.  ^a^Adverse events included with 30-day in-hospital mortality, pneumonia, septicemia, UTI.  ^b^Adjusted for all covariates listed in TABLE 1. | | | | | | |

| **Table S3** Post-stroke outcomes after matching procedure in patients with and without irregular dental scaling | | | | | | |
| --- | --- | --- | --- | --- | --- | --- |
|  | No DS (N=77786) | | IDS (N=77786)‡ | | Risk of outcomes | |
| Post-stroke outcomes | Events | % | Events | % | OR | (95% CI)* |
| 30-day in-hospital mortality | 3642 | 4.7 | 3259 | 4.2 | 0.88 | (0.84-0.93) |
| Pneumonia | 3899 | 5.0 | 2981 | 3.8 | 0.75 | (0.71-0.78) |
| Septicemia | 1683 | 2.2 | 1213 | 1.6 | 0.71 | (0.66-0.77) |
| Urinary tract infection | 4661 | 6.0 | 4013 | 5.2 | 0.85 | (0.81-0.89) |
| Stay in intensive care unit | 20400 | 26.2 | 19584 | 25.2 | 0.92 | (0.90-0.95) |
| Medical expenditure, USD^†^ | 2199±3093 | | 2147±3110 | | p=0.0010 | |
| Length of hospital stay, days^†^ | 9.6±9.0 | | 9.2±8.8 | | p<0.0001 | |
| CI, confidence interval; DS, dental scaling; IDS, irregular dental scaling; OR, odds ratio.  *Adjusted for age, sex, low income, types of stroke, hypertension, diabetes, mental disorders, ischemic heart disease, chronic obstructive pulmonary disease, hyperlipidemia, heart failure, Parkinson’s disease, liver cirrhosis, and renal dialysis.  †Mean±SD; regular dental scaling was associated with length of hospital stays (beta = -0.46, p<0.0001) and medical expenditure (beta = -51.9, p=0.0004) after adjusted all above factors in the multiple linear regressions.  ‡Irregular dental scaling was defined as people who had 1, 2, or 3 visits of dental scaling within 24 months before stroke admission. | | | | | | |
